# Cut-C: cleavage under tethered nuclease for conformational capture

**DOI:** 10.1186/s12864-019-5989-2

**Published:** 2019-07-29

**Authors:** Takashi Shimbo, Machika Kawamura, Edward Wijaya, Eiichi Takaki, Yasufumi Kaneda, Katsuto Tamai

**Affiliations:** 1 Department of Stem Cell Therapy Science, Graduate School of Medicine, Osaka University, Suita, Osaka, 5650871 Japan; 2 StemRIM Co., Ltd., Ibaraki, Osaka, 5670085 Japan; 3 Division of Gene Therapy Science, Graduate School of Medicine, Osaka University, Suita, Osaka, 5650871 Japan

**Keywords:** Chromosome conformation, Cut-C, Gene regulation, Next-generation sequencing

## Abstract

**Background:**

Deciphering the 3D structure of the genome is essential for elucidating the regulatory mechanisms of gene expression in detail. Existing methods, such as chromosome conformation capture (3C) and Hi-C have enabled the identification of novel aspects of chromatin structure. Further identification of protein-centric chromatin conformation is enabled by coupling the Hi-C procedure with a conventional chromatin immunoprecipitation assay. However, these methods are time-consuming and require independent methods for validation.

**Results:**

To simultaneously identify protein-centric chromatin conformation and target protein localization, we have developed Cut-C, a method that combines antibody-mediated cleavage by tethered nuclease with chromosome conformation capture to identify chromatin interactions mediated by a protein of interest. Applying Cut-C to H3K4me3, a histone modification enriched at active gene promoters, we have successfully identified chromatin loops mediated by H3K4me3 along with the genome-wide distribution of H3K4me3. Cut-C also identified chromatin loops mediated by CTCF, validating the general applicability of the method.

**Conclusions:**

Cut-C identifies protein-centric chromatin conformations along with the genome-wide distribution of target proteins using simple procedures. The simplified protocol will improve the efficiency of analysing chromatin conformation using precious materials, such as clinical samples.

**Electronic supplementary material:**

The online version of this article (10.1186/s12864-019-5989-2) contains supplementary material, which is available to authorized users.

## Background

Resolution of the three dimensional (3D) conformation of chromatin is essential for understanding the regulatory mechanisms involved in gene expression [1]. Chromatin conformation signatures determine the lineage-specific differentiation of cells with identical genomes. Understanding how multiple proteins, such as CTCF, regulate chromatin conformation is essential to fully appreciate the complexity of gene expression regulation [2].

Revolutionary techniques, such as chromosome conformation capture (3C) and its sequencing version, Hi-C, have enabled the understanding of numerous aspects of chromatin conformation, including gene loops, promoter-enhancer loops, and topologically associated domains [3, 4]. Further derivatives of Hi-C, such as ChIA-PET and HiChIP, have been developed to analyse the protein-centric chromatin conformation by coupling the Hi-C procedure with the conventional chromatin immunoprecipitation assay (ChIP) [5–7]. Genome wide localization information of a target protein along with the chromatin conformation mediated by the protein is essential to uncover protein-centric chromatin conformation [8, 9]. However, because these techniques are mainly focused on identifying chromatin conformation, independent ChIP-seq experiments (or equivalents), which are generally time-consuming, need to be performed to precisely map the genome wide localization of target proteins. Therefore, an improved technique to simultaneously identify the chromatin conformation and precisely map target proteins using simple and robust procedures is essential. In this study, we aimed to develop a method that combines antibody-mediated cleavage by tethered nuclease with chromosome conformation capture to identify chromatin interactions mediated by a protein of interest along with the genome-wide distribution of the target proteins.

## Results and discussion

**Fig. 1:**
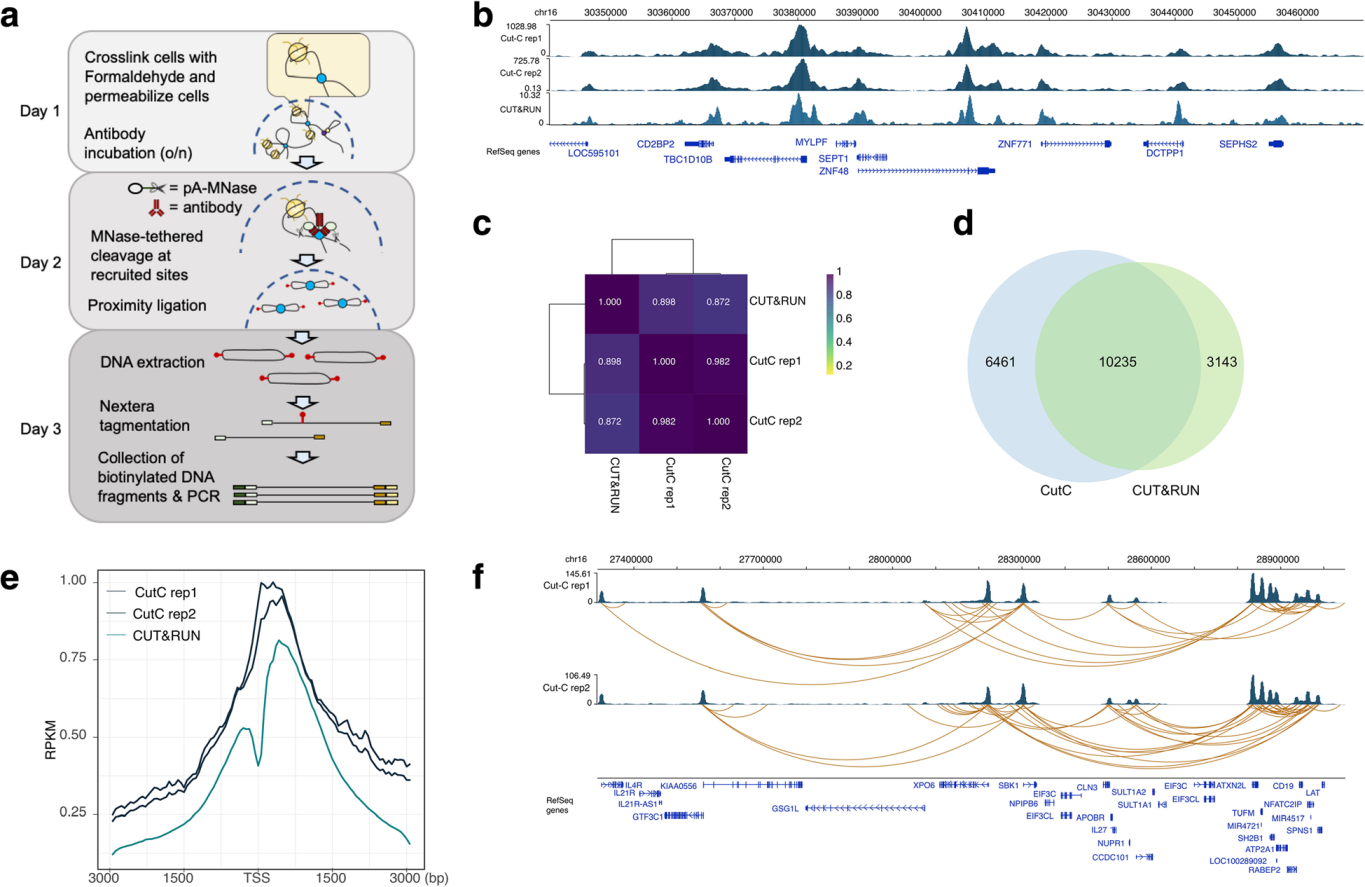
Analysis of protein localization and chromatin loops by Cut-C. **a** Schematic representation of the Cut-C procedure. **b** Representative screenshot for H3K4me3 Cut-C data. H3K4me3 CUT&RUN data are also presented for validation. **c** Pearson correlation among H3K4me3 Cut-C (two biological replicates) and CUT&RUN data. **d** Peak overlap among H3K4me3 Cut-C (two biological replicates) and CUT&RUN data. **e** Reads enrichment at promoter regions. Relative distance to transcription start sites (TSSs) are plotted. **f** Exemplar shot for chromatin interactions detected by H3K4me3 Cut-C

To simultaneously identify the chromatin conformation and precisely map target proteins, we have developed Cut-C, a method implementing the cleavage under tethered nuclease technology into Micro-C, a Hi-C derivative using micrococcal nuclease (MNase) instead of restriction enzymes, to fragment chromatin [10, 11]. In Hi-C derivatives, DNA interactions are labelled by specific cleavages (mediated by restriction enzymes, mechanical forces, or MNase) followed by proximity ligation. In contrast, in Cut-C, only a subset of the long-range DNA interactions, whose conformation is mediated by a target protein, is labelled by a spatially controlled cleavage by protein A-fused MNase, which is tethered by antibodies against a target protein, followed by proximity ligation and subsequent DNA interaction enrichment and amplification by a tagmentation-based library preparation method. The resulting libraries can be analysed by massive parallel sequencing in a paired-end mode. Similar to other Hi-C derivatives, paired-end tags (PETs) yield chromatin conformation data. Furthermore, as the cleavage of MNase is tightly regulated at the protein binding location, identification of cleaved sites provides a precise map for the target protein. The simple Cut-C experiment can be conducted in 3 days (Fig. 1a and Methods).

To test this method, we performed Cut-C for active histone marks using anti-H3K4me3 antibody in a commonly used human cell line, HEK293T (Additional file 1). To assess reproducibility, we prepared Cut-C libraries with two biological replicates (10 million cells) and obtained 206,471,443 and 139,158,639 paired-end sequencing reads, respectively. We first assessed the power of Cut-C to identify the genome wide localization of a target protein; we analysed Cut-C data (paired-end data were decoupled and analysed as single-end data, in general) with standard ChIP-seq analysis pipeline. To evaluate the localization identified by Cut-C, we performed CUT&RUN with anti-H3K4me3 antibody in HEK293T cells. A representative screenshot of the genome browser shows that Cut-C provides protein localization information with high quality in good agreement with the CUT&RUN data (Fig. 1b) with high overall correlation (Fig. 1c). To further validate the enrichment specificity, ChIP peaks were called using MACS2. We identified 16,696 peaks with Cut-C data (two replicates combined) and 13,378 peaks using CUT&RUN data, and the identified peaks show a high degree of overlap (> 61%) (Fig. 1d). We also confirmed that the Cut-C reads were strongly enriched around transcription start sites (TSSs), reflecting the H3K4me3 modification pattern (Fig. 1e), suggesting that Cut-C can provide protein localization information.

To assess the chromatin interactions identified by Cut-C, we analysed the data using the HiC-Pro pipeline [12]. Approximately 59% of the total sequencing reads were informative and valid paired end tags (PETs), as defined by HiC-Pro in the Cut-C data for H3K4me3 in HEK293T cells (Additional file 2). We detected 206,471,443 and 139,158,639 total informative PETs in the two biological replicates, respectively. Of those, majority of the identified PETs was intra-chromosomal PETs and only about 5% was consisted by the inter-chromosomal PETs (Additional file 2). Although the intra-chromosomal PETs showed high consistency between the biological replicates (more than 90% overlap), but only about 13% of the inter-chromosomal PETs were overlapped (Additional file 3). Further investigations will be required to judge whether these inter-chromosomal PETs are bone fide interactions. Using the cLoop pipeline, we call loops using the identified PETs by Cut-C. We detected 98,199 (H3K4me3 replicate 1) and 131,508 (H3K4me3 replicate 2) loops (Additional file 2). The example screenshot shows the chromatin loops identified using Cut-C (Fig. 1f). The genomic distance of the identified loops showed agreement between biological replicates, validating the reproducibility of the Cut-C procedure (Additional file 4).

**Fig. 2:**
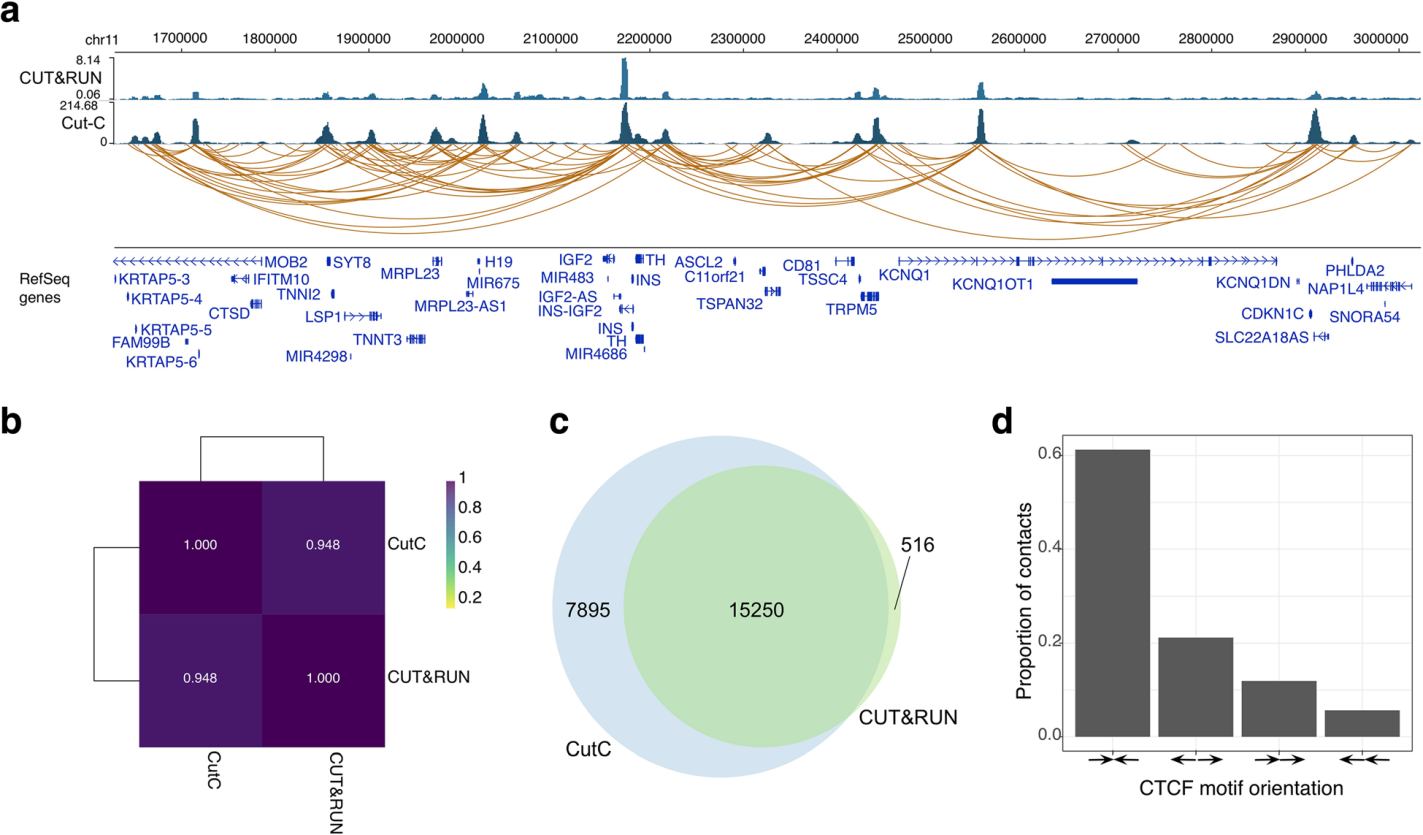
General applicability of Cut-C. **a** Exemplar shot for CTCF Cut-C. **b** Pearson correlation between CTCF Cut-C and CUT&RUN data. **c** Peak overlap between CTCF Cut-C and CUT&RUN data. **d** CTCF motif orientation in read pairs

To test general applicability, we performed Cut-C using an anti-CTCF antibody in HEK293T cells (10 million cells) and obtained 140,345,513 sequencing reads and 52,518 loops (Additional file 2). CTCF Cut-C successfully identified the chromatin loops (Fig. 2a). The example genome browser shot and Pearson correlation showed a high agreement between Cut-C and CTCF CUT&RUN data (Fig. 2a and b). We identified 23,145 and 15,766 peaks using Cut-C and CUT&RUN data, respectively, and the called peak showed a high degree of overlap (Fig. 2c). We also confirmed that Cut-C recapitulated the previously identified orientation of the CTCF motif (Fig. 2d), underlining its applicability to multiple antibodies.

Using the targeted cleavage of the antibody-tethered MNase, we successfully eliminated the ChIP step, which is time-consuming and laborious. Most methods to identify protein-centric chromatin conformation, including HiChIP, involve ChIP to enrich the target protein. Because Cut-C does not require chromatin solubilization, we could omit chromatin fragmentation by sonication, which potentially denatures epitopes [13].

## Conclusions

Here we introduced Cut-C, a simple method to delineate the protein-centric chromatin conformation and protein localization using a single experiment. By targeting H3K4me3 and CTCF, we showed that Cut-C has the potential to detect chromatin conformation. The simplified procedure of Cut-C will also facilitate the analysis of chromatin conformation in various biological samples, including patient clinical samples.

## Methods

### Cell culture and sampling

HEK293T cells were cultured in 10% FBS/ 1% penicillin-streptomycin/DMEM. Cells were trypsinized and washed with 2% FBS/PBS and 1 × 10^7^ cells were centrifuged at 300×*g* for 5 min at room temperature. Cells were fixed with 1% formaldehyde/2% FBS/PBS (1 ml) for 10 min at room temperature. Fixation was quenched with ice-cold glycine to a final concentration of 250 mM for 5 min at room temperature with gentle rocking, and on ice for 15 min with occasional mixing. Cells were centrifuged at 800×*g*, 5 min, 4 °C; washed twice with PBS; and snap-frozen with liquid nitrogen.

### Cut-C

Cells were first lysed with lysis buffer (10 mM Tris-HCl (pH 7.4), 10 mM NaCl, 3 mM MgCl_2_, 0.1% Tween 20, 0.1% IGEPAL CA-630, 0.01% Digitonin) for 30 min on ice, with occasional mixing [14–16]. Lysis was stopped by adding an equal volume of resuspension buffer (10 mM Tris-HCl (pH 7.4), 10 mM NaCl, 3 mM MgCl_2_, 0.1% Tween 20), and the cell pellet was collected by centrifugation (800×*g*) for 5 min at 4 °C. The cell pellet was washed twice with wash buffer (20 mM HEPES, 150 mM NaCl, 0.5 mM Spermidine, 1× Protease Inhibitor) [11, 16] and was resuspended in 500 μL antibody-containing buffer (20 mM HEPES, 150 mM NaCl, 0.5 mM Spermidine, 1× Protease Inhibitor, 0.02% or 0.0375% Digitonin, 2 mM EDTA). Cells were incubated overnight with 1 μg anti- H3K4me3 antibody (Abcam; ab8580) or 5 μg anti-CTCF antibody (Abcam; ab70303), followed by centrifugation at 500×*g* for 5 min at 4 °C and washed twice with 1 ml digitonin buffer (20 mM HEPES, 150 mM NaCl, 0.5 mM Spermidine, 1× Protease Inhibitor, 0.02% or 0.0375% Digitonin). The cell pellet was resuspended with digitonin buffer and incubated with empirically tested Protein A-MNase (Protein A-MNase fused protein was purified as previously described with minor modifications [17]) for 1 h at 4 °C with rotation, as previously described. MNase-tethered cleavage was performed by incubating the sample at 37 °C for 20 min. The CUT&RUN was stopped by adding EGTA to a final concentration of 2 mM and incubated at 65 °C for 10 min. The cell pellet was processed for dephosphorylation, end-chewing, end-labelling, and in situ proximity ligation of DNA fragments cleaved by tethered protein A-MNase as previously described, with minor modifications [18].

### Library preparation

Genomic DNA samples from Cut-C were purified through phenol-chloroform extraction and ethanol precipitation [18]. After air-drying, the pellet was resuspended in 50 μL Buffer EB (Qiagen) and heated at 37 °C for 15 min. The sample was then purified further using Genomic DNA Clean & Concentrator (Zymo Research) and eluted in 50 μL Buffer EB (Qiagen). The concentration of DNA was measured with Qubit 3.0 Fluorometer (Invitrogen/Life Technologies). Multiple 50 μL aliquots, consisting of 1 μL Tagment DNA Enzyme 1(Nextera DNA Library Prep Kit, Illumina), 21.5 ng or 10 ng DNA and 25 μL Tagment DNA Buffer (Nextera DNA Library Prep Kit, Illumina), were prepared for tagmentation. Tagmentation was performed at 55 °C for 10 min. DNA was extracted using Genomic DNA Clean & Concentrator (Zymo Research) and eluted with 50 μL Buffer EB (Qiagen). Ten microliters Dynabeads M-280 Streptavidin beads (Thermo Fisher Scientific) were washed and prepared. Briefly, the 50 μL chromatin sample and 50 μL bead suspension were mixed and incubated for 30 min for room temperature on a rotor. The biotin-tagged DNA fragments were collected on a magnetic stand and resuspended in 20 μL or 40 μL Buffer EB (Qiagen). PCR was performed as indicated by Buenrostro et al. (2015) [14], with minor modifications: 10 μM Nextera N7xx and S5xx 2.5 μL each (0.5 μM for each primer) (Nextera XT Index Kit v2, Illumina), KAPA HiFi HotStart ReadyMix (KAPA Biosystems) 25 μL, DNA and water to a total volume of 50 μL, with the PCR program: 72 °C, 5 min; 98 °C, 3 min; (98 °C 10 s, 63 °C 30 s, 72 °C 1 min) 5 cycles; 72 °C 1 min, 4 °C ∞. Beads were size-selected by 1.0× using AMPure XP beads (Beckman Coulter). Additional cycles were calculated by real-time qPCR. The final 50 μL PCR product was size selected by removing large DNA fragments purified by the 0.45× (22.5 μL) concentration of AMPure XP beads; the DNA fragments were collected using a 1.0× final (50 μL beads) concentration of AMPure XP beads. Size selection was repeated and DNA fragments were eluted in 13 μL of Buffer EB (Qiagen). Concentrations were measured with 1 μL sample volumes (Qubit 3.0 Fluorometer) and the remaining sample was used for Tape Station analysis (Agilent Technologies) and sequencing with NextSeq 500 (Illumina).

### CUT&RUN and library preparation

CUT&RUN analyses of H3K4me3 and CTCF localization were performed with 80,000 and 1 × 10^6^ HEK293T cells, respectively, by following the CUT&RUN protocol [11, 16] with minor modifications. Briefly, cells were centrifuged at 600×*g* for 3 min with wash buffer. Antibody incubation was performed for 2 h with the 0.02% digitonin-containing antibody buffer, which contained either 1 μg anti-H3K4me3 (Abcam; ab8580) or 1 μg anti-CTCF antibody (Abcam; ab70303) as described above. Cells were washed and resuspended with 0.02% digitonin/ wash buffer by centrifugation at 500×*g* for 5 min at 4 °C. After the addition of pA-MNase as described above, CUT&RUN was performed for 30 min on a chilled ice block and stopped by adding 2× stop buffer (4.2 ml water, 340 μL 5 M NaCl, 200 μL 0.5 M EDTA, 100 μL 0.2 M EGTA, 20 μL 5% Digitonin, 25 μL RNase A). The CUT&RUN library was generated using the Accel-NGS® 1S Plus DNA Library Kit (Swift Biosciences) and 1S Plus Combinatorial Dual Indexing Kit (Swift Biosciences). DNA fragments were size-selected by adding 1.0× ratio of AMPure Beads XP (Beckman Coulter). DNA was amplified using the following PCR program: 98 °C 30 s, (98 °C 10 s, 60 °C 30 s, 68 °C 60 s) × 5 cycles, 4 °C ∞. Additional cycles were carried out using real-time qPCR. The final PCR amplicon was size-selected using AMPure XP Beads as described above and sequenced using NextSeq 500 (Illumina).

### Bioinformatics analysis

#### Cut-C data processing

The paired-end reads for Cut-C were processed using HiC-Pro [12]. This pipeline includes read alignment mapping to the hg19 genome, with bowtie2 parameter (−-very-sensitive -L 30 --score-min L,-0.6,-0.2 --end-to-end –reorder). All parameters, except for the ligation site and minimum cis distance threshold and duplicate removal flag, were set to default. The valid interaction produced by HiC-Pro was used for quality control. The results shown in Fig. 1f and Fig. 2d are based on minimum cis distance 0 and without duplicate removal.

Loop calling was carried out using cLoops with the following parameters: -m 0 -eps 5000,10000 -minPts 2,5,3 -p 20 -w -j -s -hic -cut 2000 -plot -max_cut [19]. We also used these called loops to analyse H3K4me3 Cut-C loop frequency with regard to genomic distances between loop ends.

#### Cut-run data processing

The paired-end CUT&RUN reads were processed using TrimGalore (https://github.com/FelixKrueger/TrimGalore) with the following parameters (−stringency 5 –paired –trim1 –length 30 -q 0 –a CTGTCTCTTATACACATCT) to trim adaptor sequences and then aligned using Bowtie [20] with the following parameters (−m 1 -v 2 -S -I 0 -X 2000). Duplicated reads were removed using Picard’s MarkDuplicates (http://broadinstitute.github.io/picard/) with default settings. The reads mapped to the blacklist features as defined in the ENCODE project [21] were removed. Peak calling was performed using MACS2 with the following parameters (callpeak –nomodel –nolambda –keep-dup all). Feature intersections were done using BEDTools [22]. For correlation analysis, the read density within a peak was calculated using featureCounts [23].

#### Enrichment analysis

TSS and CTCF enrichment analysis for Cut-C and CUT&RUN were performed using *ngs.plot.r* [24]. Prior to TSS analysis, the read-pairing in the alignment (BAM) file produced by HiC-Pro in the Cut-C data was decoupled. This was required to prevent the inclusion of the region caused by cross-linking.

#### CTCF motif orientation analysis

Initially, we obtained the genome-wide CTCF motif binding location from JASPAR 2018 UCSC motif tracks (http://jaspar.genereg.net/genome-tracks/). From HiC-Pro valid interaction data, we separated the first and second anchor locations of the interacting reads. By intersecting the CTCF binding with the anchor location, we determined the motif orientation for each interacting read. Finally, the percentage of interacting reads with converging CTCF motif orientation (+ strand on 5′ end and – strand motif on 3′ end), diverging orientation (−/+), or the same orientation (+/+ or −/−) were counted and divided by the total number of interacting reads.

## Additional files

Additional file 1 Cut-C library validation. **a**, **b**. DNA size of Cut-C libraries were validated using Tapestation, H3K4me3 libraries (a) and CTCF library (b). (PNG 493 kb)

Additional file 2 Quality control data for all Cut-C data presented in this study. (XLSX 10 kb)

Additional file 3 PETs overlap between H3K4me3 Cut-C biological replicates. (XLSX 9 kb)

Additional file 4 Cut-C loop frequency for H3K4me3 with regard to genomic distances between anchors. (PNG 347 kb)

## Data Availability

All sequencing data used in this study are deposited at GEO (GSE125988).

## Abbreviations

3C: Chromosome conformation capture
ChIA-PET: Chromatin interaction analysis by paired-end tag sequencing
ChIP: Chromatin immunoprecipitation
Cut-C: Cleavage under tethered nuclease for conformational capture
MNase: Micrococcal nuclease
PET: Paired end tag
