# Supplementary figures and images for: Cut-C: cleavage under tethered nuclease for conformational capture
Source: BMC Genomics. 2019 Jul 29;20:614. doi: 10.1186/s12864-019-5989-2 (PMC6664727; doi:10.1186/s12864-019-5989-2)

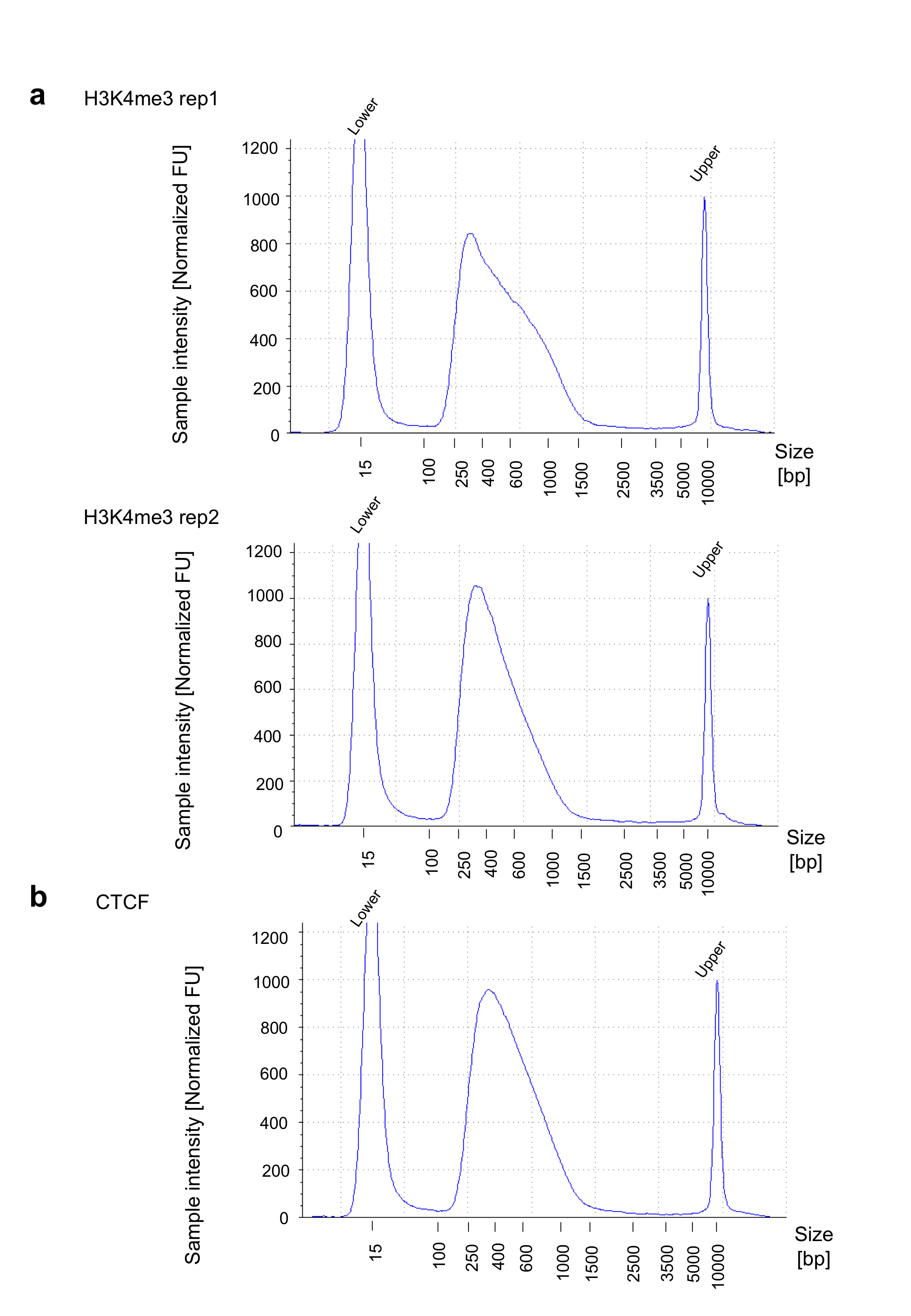

Supplement: Supplementary file 1 — Cut-C library validation. a, b. DNA size of Cut-C libraries were validated using Tapestation, H3K4me3 libraries (a) and CTCF library (b). (PNG 493 kb) [file 12864_2019_5989_MOESM1_ESM.png]

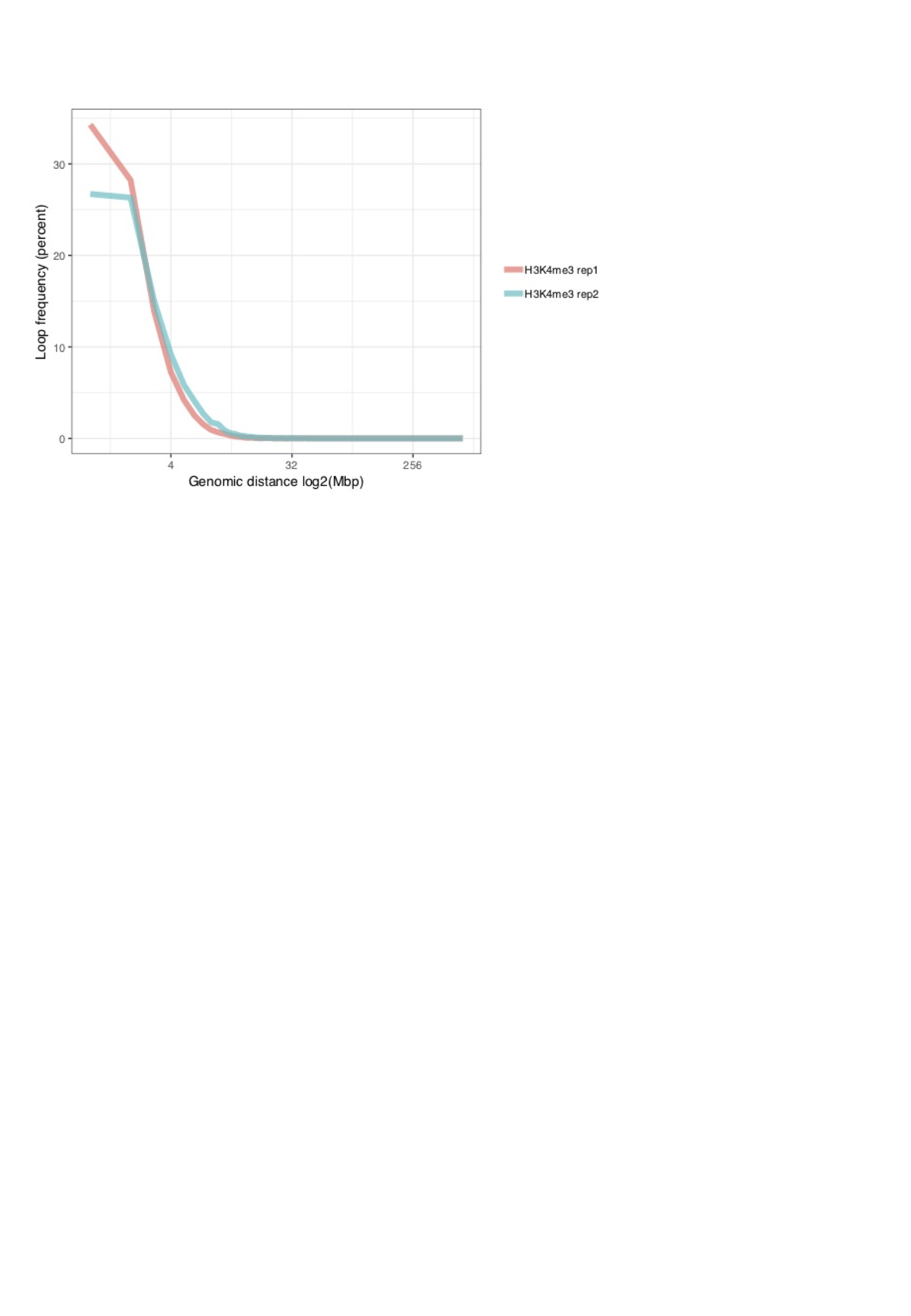

Supplement: Supplementary file 4 — Cut-C loop frequency for H3K4me3 with regard to genomic distances between anchors. (PNG 347 kb) [file 12864_2019_5989_MOESM4_ESM.png]
